# Genome- and epigenome-wide association study of hypertriglyceridemic waist in Mexican American families

**DOI:** 10.1186/s13148-016-0173-x

**Published:** 2016-01-20

**Authors:** Manju Mamtani, Hemant Kulkarni, Thomas D. Dyer, Harald H. H. Göring, Jennifer L. Neary, Shelley A. Cole, Jack W. Kent, Satish Kumar, David C. Glahn, Michael C. Mahaney, Anthony G. Comuzzie, Laura Almasy, Joanne E. Curran, Ravindranath Duggirala, John Blangero, Melanie A. Carless

**Affiliations:** South Texas Diabetes and Obesity Institute, University of Texas Rio Grande Valley School of Medicine, Brownsville, TX 78520 USA; Department of Genetics, Texas Biomedical Research Institute, San Antonio, TX USA; Department of Psychiatry, Yale University, New Haven, CT USA; Olin Neuropsychiatry Research Center, Institute of Living, Hartford Hospital, Hartford, CT USA

## Abstract

**Background:**

There is growing interest in the hypertriglyceridemic waist (HTGW) phenotype, defined as high waist circumference (≥95 cm in males and ≥80 cm in females) combined with high serum triglyceride concentration (≥2.0 mmol/L in males and ≥1.5 mmol/L in females) as a marker of type 2 diabetes (T2D) and cardiovascular disease. However, the prevalence of this phenotype in high-risk populations, its association with T2D, and the genetic or epigenetic influences on HTGW are not well explored. Using data from large, extended families of Mexican Americans (a high-risk minority population in the USA) we aimed to: (1) estimate the prevalence of this phenotype, (2) test its association with T2D and related traits, and (3) dissect out the genetic and epigenetic associations with this phenotype using genome-wide and epigenome-wide studies, respectively.

**Results:**

Data for this study was from 850 Mexican American participants (representing 39 families) recruited under the ongoing San Antonio Family Heart Study, 26 % of these individuals had HTGW. This phenotype was significantly heritable (*h*^2^*r* = 0.52, *p* = 1.1 × 10^−5^) and independently associated with T2D as well as fasting glucose levels and insulin resistance. We conducted genome-wide association analyses using 759,809 single nucleotide polymorphisms (SNPs) and epigenome-wide association analyses using 457,331 CpG sites. There was no evidence of any SNP associated with HTGW at the genome-wide level but two CpG sites (cg00574958 and cg17058475) in *CPT1A* and one CpG site (cg06500161) in *ABCG1* were significantly associated with HTGW and remained significant after adjusting for the closely related components of metabolic syndrome. *CPT1A* holds a cardinal position in the metabolism of long-chain fatty acids while *ABCG1* plays a role in triglyceride metabolism.

**Conclusions:**

Our results reemphasize the value of HTGW as a marker of T2D. This phenotype shows association with DNA methylation within *CPT1A* and *ABCG1*, genes involved in fatty acid and triglyceride metabolism. Our results underscore the importance of epigenetics in a clinically informative phenotype.

**Electronic supplementary material:**

The online version of this article (doi:10.1186/s13148-016-0173-x) contains supplementary material, which is available to authorized users.

## Background

As the global epidemic of type 2 diabetes (T2D) continues to expand, better, accurate, and cost-efficient ways to stratify risk of individuals are urgently required. In that vein, it is now fairly well established that hypertriglyceridemic waist (HTGW) is a promising and simple measurement that is clinically feasible, yet accurate for risk stratification of T2D [1–3]. HTGW is a critical link between obesity (specifically, central obesity) and T2D. The rationale for using a combination of waist circumference and fasting triglyceride levels is to partially overcome the inability of waist circumference to discriminate between subcutaneous and visceral adiposity; raised serum triglycerides in the presence of increased waist circumference are more likely to indicate visceral rather than subcutaneous fat accumulation [4]. The last few years have seen a dramatic increase in studies around the world that have reported the use and importance of HTGW in prediction of T2D and cardiovascular risk [5–12].

Considering the strong genetic basis of T2D, waist circumference, and serum triglyceride levels, it is likely that there is also a strong genetic contribution to HTGW. Several studies have shown the possible involvement of *HNF1A* variants, epistatic interactions with genes involved in the very low density lipoprotein pathway and variation within *ADIPOQ* to be associated with HTGW [13–16]. Generally, however, replicative literature support for these observations from large, population based studies has been lacking. Moreover, given the possibility of a significant environmental influence (e.g., through diet and physical activity) on both waist circumference and triglyceride levels [11, 17, 18], it is conceivable that the HTGW phenotype may be epigenetically modifiable. Although recent elegant studies have shown promising results for the association of DNA methylation separately with waist circumference [19–21] and triglyceride levels [22–24], to our knowledge, currently, there are no studies on the possible epigenetic role in the inter-individual variability in HTGW.

In this study, we investigated both the genetic and epigenetic basis of HTGW in Mexican Americans, a minority population in the USA who are at high risk for both obesity and T2D [25–27]. To enhance our power to detect genetic and epigenetic effects, we conducted this study in pedigreed individuals representing large, extended families, recruited in the San Antonio Family Heart Study [28, 29]. Using genome- and epigenome-wide approaches, we show here an association between HTGW and T2D in Mexican Americans and identify three CpG sites in *CPT1A* and *ABCG1* that are significantly and independently associated with the HTGW phenotype.

## Results

### Study participants

We studied 850 pedigreed Mexican Americans (representing 39 extended families), who were predominantly middle aged (mean age 46.75 years, standard deviation 14.54 years) and 63 % female. At the time of assessment 21 % of individuals had T2D (fasting glucose ≥7 mmol/L), another 17 % had impaired fasting glucose (fasting serum glucose between 5.55 mmol/L and 7 mmol/L), 56 % were obese (body mass index ≥30 kg/m^2^), and 32 % had hypertension (systolic blood pressure >140 mmHg and/or diastolic blood pressure >90 mmHg, or already receiving antihypertensive treatment). A total of 14, 16, and 24 % of participants were receiving lipid-lowering, anti-diabetic and anti-hypertension medications, respectively. The prevalence of central obesity (waist circumference ≥90 cm for males or ≥85 cm for females) was very high (88 %) in our sample but the prevalence of hypertriglyceridemia (≥2.0 mmol/L for males and ≥1.5 mmol/L in females) was comparatively lower (28 %). There were a total of 223 (26 %) participants with HTGW, with a higher prevalence in females (31 %) compared to males (18 %). To ensure that the potential association of genetic and epigenetic variants with HTGW is not confounded by the presence of comorbidities, we used robust statistical models that accounted for the simultaneous presence of comorbidities (systolic and diastolic blood pressure and presence of diabetes and obesity) in our analyses.

There was a significant phenotypic correlation between waist circumference and triglycerides in the study participants (*ρ*_P_ = 0.19, *p* = 6.8 × 10^−7^). However, results obtained using bivariate trait analyses showed that this phenotypic correlation was primarily because of shared environmental influences (*ρ*_E_ = 0.32, *p* = 0.0011) rather than due to shared genetic influences (*ρ*_G_ = 0.07, *p* = 0.5898). We therefore conducted an investigation into the potential genetic and epigenetic basis of HTGW.

### Association of HTGW with T2D

We first tested the hypothesis that HTGW is an independent determinant of T2D in our sample of Mexican American families. For this, we tested the association of HTGW with four T2D-related phenotypic traits (T2D, fasting blood glucose (FBG), fasting plasma insulin (FPI), and homeostatic model of assessment-insulin resistance (HOMA-IR)). After adjusting for clinically relevant confounders, we found that HTGW was significantly and independently associated with all four traits related to T2D (Table 1). The polygenic regression coefficients, when transformed to odds ratios, indicate that study participants with HTGW have 3.16 times higher odds (as compared to those without HTGW) of T2D (95 % CI 2.00–4.93).

**Table 1:** Independent association of HTGW with T2D-related traits in San Antonio Family Heart Study participants

	Trait^a^			
	T2D	FBG	FPI	HOMA-IR
Model specification				
Type of trait	Discrete	Continuous	Continuous	Continuous
Transformation	Liability	Inverse normalization	Inverse normalization	Inverse normalization
Covariate set^b^	A	B	B	B
Association with HTGW				
*b* (95 % CI)	0.65 (0.39–0.90)	0.30 (0.17–0.43)	0.16 (0.02–0.29)	0.26 (0.13–0.38)
*P*	3.7 × 10^−7^	1.2 × 10^−5^	0.0236	9.8 × 10^−5^
Association of HTGW and its components estimated through an interactive model				
High TG and WC				
*b* (95 % CI)	0.87 (0.31–1.43)	0.45 (0.22–0.68)	0.32 (0.08–0.56)	0.45 (0.22–0.67)
*P*	0.0021	0.0001	0.0079	8.0 × 10^−5^
High WC only				
*b* (95 % CI)	0.25 (0.13-0.37)	0.16 (0.04-0.29)	0.17 (−0.18-0.52)	0.20 (0.00-0.39)
*P*	5.9 × 10^−5^	0.0081	0.3472	0.0399
High TG only				
*b* (95 % CI)	0.22 (−1.66–2.09)	0.24 (−1.79–2.28)	0.28 (−0.86–1.43)	0.37 (−0.62–1.37)
*P*	0.8194	0.8160	0.6284	0.4612

*HTGW* hypertriglyceridemic waist, *TG* Triglycerides, *WC* Waist circumference, *b* regression coefficient, *p* significance value

^a^ *T2D* type 2 diabetes, *FBG* fasting blood glucose, *FPI* fasting plasma insulin, *HOMA-IR* homeostatic model of assessment—insulin resistance

^b^ *Covariate set A*, age, age^2^, sex, age × sex interaction, age^2^ × sex interaction, body mass index, systolic and diastolic blood pressure, use of anti-lipid and anti-hypertensive medications; *Covariate Set B* age, age^2^, sex, age × sex interaction, age^2^ × sex interaction, body mass index, systolic and diastolic blood pressure, use of anti-lipid, anti-hypertensive, and anti-diabetic medications

We next evaluated whether the presence of both high waist circumference and hypertriglyceridemia is more strongly associated with T2D-related traits than either of these factors alone. For this, we used interactive polygenic regression models, the results of which are shown in Table 1. For all four traits studied, the regression coefficients for the combination of high waist and hypertriglyceridemia were statistically significantly associated with the trait in question. With the exception of T2D, the regression coefficients for the combination of high waist circumference and hypertriglyceridemia were substantially larger as compared to those for high waist circumference or hypertriglyceridemia alone. These results indicate that in the context of T2D-related phenotypes, HTGW is likely to be strongly associated with the underlying pathology of T2D and may therefore be an important T2D risk assessment tool.

### Heritability of HTGW

We next estimated the heritability of HTGW using polygenic regression that accounted for familial relationships. Even after accounting for age and sex (and their first and second order interactions), use of lipid lowering drugs, use of anti-hypertensive medications, systolic blood pressure, diastolic blood pressure, and presence of diabetes and obesity, we found that the heritability of HTGW was 0.52 (SE 0.12, *p* = 1.1 × 10^−5^).

### Genome-wide association analysis of HTGW

**Fig. 1:**
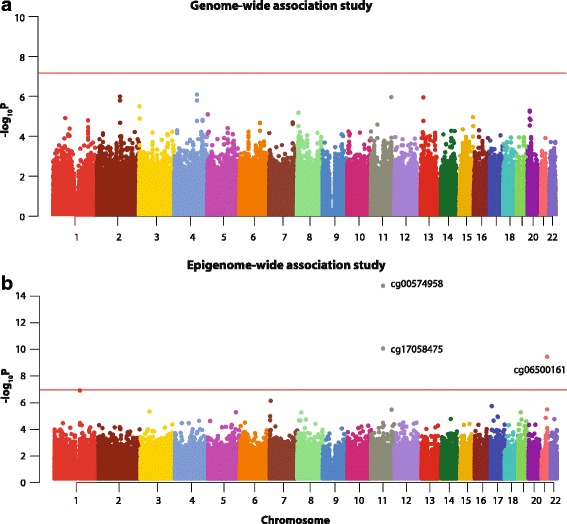
Manhattan plots showing the genome-wide association of DNA sequence variants and epigenome-wide association of DNA methylation with liability of HTGW. **a** Genome-wide association study. Genome-wide significance is indicated by the *red horizontal line* and chromosomal locations are *color-coded*. Results are from polygenic regression models that accounted for age, age^2^, sex, age × sex interaction, and age^2^ × sex interaction, the top four principal components quantifying ancestry-based population admixture, and use of anti-lipid, anti-hypertensive and anti-diabetic medications. **b** Epigenome-wide association study. Epigenome-wide significance is indicated by the *red horizontal line*. Significantly associated CpG sites are labeled and chromosomal locations are *color-coded*. Results are from polygenic regression models adjusted for age, age^2^, sex, age × sex interaction, and age^2^ × sex interaction, Illumina Sentrix® ID and Sentrix® position (to account for batch effects), estimated cell counts, and use of anti-lipid, anti-hypertensive and anti-diabetic medications. Q-Q plots corresponding to Panel A and B are shown in Additional file 2: Figures S1 and S2, respectively

Results of the association between each of the 728,175 single nucleotide polymorphisms (SNPs) and liability of HTGW are shown in Fig. 1a, with details provided in Additional file 1: Table S1. The Q-Q plot for these analyses is shown in Additional file 2: Figure S1. There was no evidence of a systematic inflation of significance values (genomic inflation factor = 1.0251). After correcting for clinically relevant confounders as well as the top four principal components that reflected underlying population admixture, we did not find any statistically significant associations between genetic variants and liability of HTGW after correcting for multiple comparisons by controlling for a false discovery rate (FDR) of 5 %. We observed that even though clusters of SNP-HTGW association were visible on chromosomes 2 (intergenic), 4 (flanking the 3’ untranslated region of the *LOC391698* locus), 13 (flanking the 5’ untranslated region of *RFC3*), and 20 (flanking the 3’ untranslated region of the *LOC441940* locus), none of these associations reached genome-wide significance.

### Epigenome-wide analysis of HTGW

Of the 485,577 interrogated loci on the array, association analyses using polygenic regression models could be run without convergence failure on a total of 458,716 (94.5 %) autosomal CpG sites. Of these, 1385 (0.30 %) probes had detection *p* values >0.01 in >5 % of the study participants. We therefore excluded these probes from further analyses. Thus, our study included analysis on a total of 457,331 CpG sites. Full results of the association analyses between DNA methylation at each CpG site and liability of HTGW are shown graphically in Fig. 1b and provided in detail in Additional file 3: Table S2. The genomic inflation factor for these analyses was 0.6152 indicating a lack of genomic inflation of significance values (Q-Q plot for these analyses is shown in Additional file 2: Figure S2). After correcting for the clinical confounders as well as for multiple testing (FDR < 0.05), we found three CpG sites (cg00574958, cg17058475, and cg06500161, Fig. 1b and Table 2) to be significantly associated with HTGW. The top two sites were in the 5’ UTR of *CPT1A* while the third site was in *ABCG1*. It is also worth mentioning that the CpG site cg19693031 in the 3’ UTR of *TXNIP* was only marginally non-significant (FDR-corrected *p* = 0.0563, Additional file 3: Table S2). We previously found no evidence for the influence of genetic variation (±50 kb, assessed by SNP microarrays) on any of the CpG sites whose methylation levels were associated with HTGW [30]. Similarly, we had previously used whole-genome sequencing of a subset of our cohort (*n* = 197) to test for associations between probe-based SNPs and DNA methylation levels [30]. In this cohort subset, the mutant allele for rs78442314 within the *CPT1A* probe for cg00574958 was not present, and while the mutant allele for rs9982016 within the *ABCG1* probe for cg06500161 was present in 2.79 % of the cohort subset, it was not significantly associated with DNA methylation levels detected by the probe (*p* = 0.1895). Together, this suggests that the significant associations seen between DNA methylation levels and HTGW are not driven by known sequence variation.

**Table 2:** Characteristics of the CpG sites significantly associated with HTGW

Characteristic	cg00574958	cg17058475	cg06500161
Chromosome	11	11	21
Coordinate	68607622	68607737	43656587
Gene symbol	*CPT1A*	*CPT1A*	*ABCG1*
Gene context	5’UTR	5’UTR	Body
Relation with CpG Island	North shore	North shore	South shore
Presence of SNP within probe	Yes	No	Yes
Minor allele frequency of probe SNP in cohort subset	0.0000	-	0.0279
Probe SNP ➔ Methylation association in cohort subset	-	-	Not significant
Cross-reactivity	None	None	None
Heritability	0.21	0.37	0.47
*p* heritability	2.7 × 10^−5^	3.5 × 10^−10^	2.2 × 10^−15^
Median β			
HTGW	0.0831	0.0887	0.5831
No HTGW	0.0929	0.0993	0.5667
Association with HTGW^**ab**^			
*b*	−0.48	−0.44	0.36
*p*	1.7 × 10^−15^	8.7 × 10^−11^	3.7 × 10^−10^
*q*	7.9 × 10^−10^	4.0 × 10^−5^	1.7 × 10^−4^
Specificity of association with HTGW^**b**^			
*b*	−0.46	−0.40	0.32
*p*	1.3 × 10^−12^	3.4 × 10^−8^	1.8 × 10^−7^
Association with HTGW and its components estimated through an interactive model			
High TG and WC			
*b*	−0.71	−0.53	0.73
*P*	2.3 × 10^−7^	0.0002	5.3 × 10^-8^
High WC only			
*b*	−0.22	−0.25	0.27
*P*	5.7 × 10^−5^	0.0815	0.0002
High TG only			
*b*	−0.20	0.05	0.47
*P*	0.7969	0.2690	0.5328
Association with T2D that is mediated through HTGW^c^			
*b*	−0.16	−0.16	0.15
*P*	0.0223	0.0180	0.0086

*b*, *p*, and *q* indicate regression coefficient, nominal significance value, and significance level corrected for multiple testing (after correction for genomic inflation), respectively

^a^Association between DNA methylation and HTGW after adjusting for age, age^2^, sex, age × sex interaction, and age^2^ × sex interaction, Illumina Sentrix® ID and Sentrix® position (to account for batch effects), use of anti-lipid, anti-hypertensive and anti-diabetic medications and cellular heterogeneity

^**b**^Association between DNA methylation and HTGW after adjusting for age, age^2^, sex, age × sex interaction, and age^2^ × sex interaction, Illumina Sentrix® ID and Sentrix® position (to account for batch effects), use of anti-lipid, anti-hypertensive and anti-diabetic medications, cellular heterogeneity, presence of type 2 diabetes, obesity (body mass index ≥ 30 Kg/m^2^), systolic and diastolic blood pressure

^c^Mediation estimated using Sobel’s parameter [32]. All regression models were adjusted for age, age^2^, sex, age × sex interaction, and age^2^ × sex interaction, Illumina Sentrix® ID and Sentrix® position (to account for batch effects), use of anti-lipid, anti-hypertensive and anti-diabetic medications, and cellular heterogeneity

**Fig. 2:**
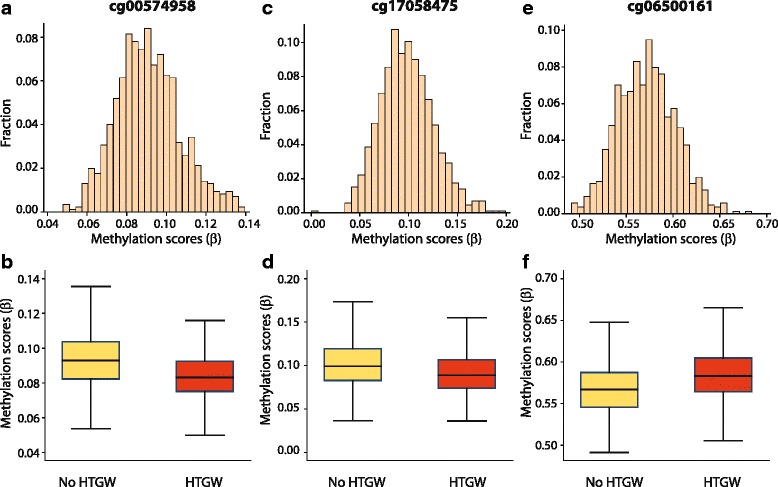
Distribution of DNA methylation scores for the significantly associated CpG sites in the study participants. Panels **a**, **c,** and **e** show histograms based on all study participants while panels **b**, **d,** and **f** show box plots for the corresponding CpG site in those with (*red boxes*) or without (*yellow boxes*) HTGW

Notably, the median *β* values (Table 2) at both CpG sites near *CPT1A* were only slightly smaller in participants with HTGW as compared to those without HTGW (1.0 % for cg00574958 and 1.1 % for cg17058475). Conversely, the median methylation levels were 1.6 % higher in individuals with HTGW as compared to those without for the CpG site near the *ABCG1*. These results indicate that seemingly small changes in DNA methylation around the *CPT1A* and *ABCG1* loci are associated with different clinical risk profiles. To determine whether the observed associations were driven by a small subset of individuals with extreme values of *β*, we studied their distribution in the study participants. We observed that there was no marked skew in the distribution of methylation at any of the three significantly associated sites (Fig. 2a, c, and e). A comparison of the methylation values categorized on the basis of presence or absence of HTGW also suggested that the observed associations were unlikely to be a result of skewed observations (Fig. 2b, d. and f). It was noteworthy that the DNA methylation levels at all of these sites were significantly heritable (Table 2; based on data from [30]). We also observed that none of the three probes were previously reported to be cross-reactive by Chen et al. [31].

To determine the specificity of association of the three CpG sites with HTGW, we further adjusted for presence of type 2 diabetes, obesity, and systolic and diastolic blood pressures. We found that there was a negligible loss in the strength of association of the three sites with HTGW after accounting for the confounding comorbidities (Table 2). Using an interactive model, we tested for association between HTGW, high waist circumference (with normal triglyceride levels), or high triglyceride levels (with normal waist circumference), and DNA methylation at each of the sites. For each CpG site, we found the strongest association to be with the HTGW phenotype. We also examined whether the strategy to include medication use as a covariate in the regression models was confounding the associations. For this, we ran two sets of models for each of these sites, one ignoring concurrent medication use and the second restricting the analyses to only those individuals who were not receiving any medication. The results of these analyses (see Additional file 4: Table S3) showed that all associations remained significant irrespective of the strategy used to account for medication use. We had previously found these sites to be associated with T2D [30], and next considered whether this is mediated through their association with HTGW. For this, we used Sobel’s method of estimating mediation [32] and found that the mediation parameter was statistically significant for each of these three sites. Together, these results show that not only are these three CpG sites strongly and independently associated with HTGW, but it is likely that the association of these sites with T2D is partly but significantly explained through HTGW.

We further considered whether the three significantly associated CpG sites contain redundant statistical information. For this, we ran a series of four models (Table 3), in which each significant CpG site was added to the polygenic regression model in a forward stepwise manner. For each step, we estimated the Kullback–Leibler *R*^2^ (K-L *R*^2^) as a measure of information content. We observed that the addition of each CpG site was associated with a statistically significant improvement in the K-L *R*^2^ statistic. The final model that included the clinical covariates along with all three CpG sites accounted for 19.67 % of the inter-individual variability in HTGW (Table 3). Considering that the base model shown in Table 3 accounted for 10.15 % of HTGW variability, our results indicate that the top three significantly associated CpG sites accounted for an additional 9.52 % of the inter-individual variability in HTGW. Together, these results show that the three sites were significantly, specifically, and independently associated with the HTGW phenotype.

**Table 3:** Improvement in Kullback-Leibler *R* ^2^ by including CpG sites significantly associated with HTGW^a^

Model	K-L *R* ^2^	∆K-L R^2^	*P*
Base^b^	0.1015	-	-
Base + cg00574958	0.1284	0.0269	2.8 × 10^−15^
Base + cg00577958 + cg17058475	0.1728	0.0444	0.0302
Base + cg00574958 + cg17058475 + cg06500161	0.1967	0.0239	2.1 × 10^−6^

^a^Analyses show the improvement in K-L *R* ^2^ statistic for a model compared to the preceding model

^b^Included following covariates: age, age^2^, sex, age × sex interaction, and age^2^ × sex interaction, Illumina Sentrix® ID and Sentrix® position (to account for batch effects), use of anti-lipid, anti-hypertensive and anti-diabetic medications, and cellular heterogeneity

### Validation of HumanMethylation450 BeadChip array by pyrosequencing

**Fig. 3:**
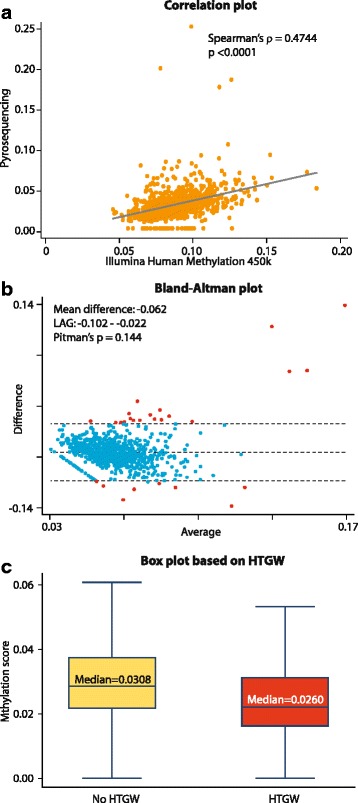
Agreement between the results of HumanMethylation450 BeadChip array and pyrosequencing for the cg00574958 CpG site. **a** Correlation scatter plot. **b** Bland-Altman plot of the difference in the measurements of the two methods (ordinate) versus the mean (abscissa). Limits of agreement (LAG) are shown pictorially using dashed *horizontal lines*. Samples that fall outside the LAG are colored *red*. **c** Distribution of the DNA methylation as measured by pyrosequencing in individuals with (*red box*) and without (*yellow box*) HTGW

We validated the measurements made by the HumanMethylation450 BeadChip array by pyrosequencing. We have previously demonstrated that for the cg06500161 CpG site within *ABCG1*, there was a significant concordance between the results obtained by HumanMethylation450 BeadChip array and by pyrosequencing (Spearman’s *ρ* = 0.42, *p* < 0.0001) [30]. Here, we demonstrate that a significant correlation also exists between the results of these two techniques for the most significantly associated CpG site in *CPT1A*, cg00574958. We observed a statistically significant correlation between the levels of methylation measured by the microarray and by pyrosequencing (Spearman’s *ρ* = 0.47, *p* < 0.0001; Fig. 3a). In general, the microarray reported inflated levels of DNA methylation compared to that measured by pyrosequencing. Therefore, we formally tested the agreement between these two methods by using Bland-Altman plot analysis (Fig. 3b). We found that on average, pyrosequencing reported methylation levels about 6.2 % less than those of the microarray. However, the limits of agreement between these two methods ranged from −0.102 to −0.022 and only 27 (~3.2 %) samples lay outside these limits (Fig. 3b, dots colored red). Pitman’s test of unequal variances was also not significant (*p* = 0.144). Thus, our results indicate that there was a moderate correlation and a very good agreement between the results obtained by microarray and pyrosequencing techniques. Consistently, we also noticed that the median DNA methylation measured by pyrosequencing was lower in individuals with HTGW as compared to those without HTGW (Fig. 3c). Further, when we ran polygenic regression models and adjusted for the same covariates as mentioned in Model 1, Table 2, we found that the regression coefficient for pyrosequencing-based methylation at cg00574958 was −0.28 (*p* = 4.4 × 10^−7^). Thus, our results using the HumanMethylation450 BeadChip array were reproducible and in agreement with those derived using pyrosequencing.

## Discussion

Our study reports several novel findings: (1) the prevalence of HTGW in our sample of Mexican Americans is ~26 %; (2) HTGW is a significant independent predictor of T2D as well as fasting glucose, insulin levels and insulin resistance; (3) HTGW is significantly heritable; (4) DNA methylation within the 5’UTR region of *CPT1A* and in the gene body of *ABCG1* is significantly associated with HTGW, independent of related comorbidities; (5) methylation at the top three significantly associated CpG sites (in *CPT1A* and *ABCG1*) together account for 9.52 % of the variability of HTGW, which is unlikely to be due to surrounding sequence variants. We also observed that seemingly small changes in DNA methylation levels (<2 % at all three identified CpG sites) are associated with HTGW. This finding implies that DNA methylation levels may be regulated with considerable plasticity and that small departures from such plasticity may have implications in disease pathogenesis through altered gene expression. It is important to consider these observations in the light of existing literature, study limitations, and clinical implications.

Using a previously established definition of HTGW [5, 33–36], our estimate of the prevalence of HTGW is within the range of 12.1–35 % prevalence previously reported by various studies across the world [6, 8, 37–41], and is comparable to that reported from a nationally representative US sample [42]. However, within our cohort, the prevalence of high waist circumference exceeded the prevalence of hypertriglyceridemia, which raises the possibility that in our study population, there was higher likelihood of accumulation of subcutaneous rather than visceral adipose tissue. Interestingly, such a finding has been confirmed with computerized tomography studies in Mexican American women [43]. This hypothesis needs to be tested and confirmed in future studies.

HTGW remains a largely understudied phenotype and neither its genetic nor epigenetic basis is known. Our study provides two initial observations in this regard. First, it reconfirms the association between HTGW and T2D-related traits [3, 7, 8, 41, 44, 45], which has previously been observed in other populations, and we now replicate in Mexican American families. Second, estimation of heritability is often considered an essential first step towards understanding the genetic basis of a phenotypic trait, and we find that HTGW is indeed significantly heritable (*h*^2^*r* = 0.52). These results advocate a need for the HTGW phenotype to be intensively investigated from a genetic perspective. It should be noted that since HTGW captures two components of the metabolic syndrome (central obesity and dyslipidemia), this phenotype is likely to be associated with coexisting conditions like hypertension, hyperglycemia, insulin resistance, and type 2 diabetes in epidemiological studies. In this study, we used robust statistical models to account for the presence of these comorbidities, and we suggest that future studies on the HTGW phenotype also be carefully designed so as to understand the true associations, not confounded by comorbidities.

We did not observe any genome-wide statistically significant associations between SNPs and HTGW. To our knowledge, this is the first genome-wide association study examining HTGW. Genome-wide association analyses have identified at least 36 loci associated with triglyceride levels, including prominent replicable associations in well implicated lipid genes *LPL*, *APOA1*, *LIPC*, and *CETP* [46–49]. Conversely, only a few loci have been implicated in waist circumference via genome-wide association studies, namely *MCR4*, *TFAP2B*, *MSRA*, *NRXN3*, and *MAP3K1*, although in most cases, these loci have not been replicated [50–54]. Our genome-wide association study of HTGW did not establish any of the previously identified loci for either triglyceride levels or waist circumference, and our most significant loci were on chromosomes 2, 4, 11, 13, and 20. While these loci may represent novel determinants of HTGW, it should be remembered that given the small effect sizes that specific genotypes typically inflict upon complex phenotypes, it is likely that our cohort is underpowered to detect such effects.

Although we did not find any evidence for association between sequence variation and HTGW in our cohort, we identified three CpG sites whose DNA methylation levels were associated with HTGW, and these associations were largely independent of other metabolic syndrome phenotypes or DNA sequence variation. Our most striking observation is the highly significant association between the *CPT1A*-related CpG sites and HTGW, which was independent of related phenotypes (blood pressure, obesity and T2D). Carnitine palmitoyltransferase acts at the outer mitochondrial membrane to escort long-chain fatty acids into the mitochondria for *β* oxidation [55]. The *CPT1A* gene that regulates the expression of this enzyme is 60 kb long and contains 20 exons, and is located on chromosome 11 [56, 57]. It is regulated by *PPARA*, a nuclear transcription factor that plays a critical role in the oxidation of fatty acids [58, 59]. Considering this molecular nexus, the role of *CPT1A* in triglyceride metabolism is now well established. However, the role of this gene in waist girth is less obvious. Two population based studies [24, 60] have shown association of sequence variants in *CPT1A* with various indices of obesity including waist circumference, but the exact mechanism by which *CPT1A* might contribute to altered adiposity remains unknown. We cannot comment on the precise mechanisms involved, but this opens up a possible and interesting thread of research for future studies. Our results are in line with the emerging evidence that methylation in the promoter region of *CPT1A* and specifically at the cg00574958 site is associated with several aspects of triglyceride biochemistry including associations with lipoprotein sub-fractions, hypertriglyceridemia, and the effect of lipid lowering drugs [22, 23, 61].

Our results indicate that the other gene that might play a role in HTGW by way of altered methylation levels is the ATP binding cassette G1 (*ABCG1*) gene. There is increasing evidence to show that this gene plays an important role in triglyceride metabolism [62–65]. For example, a recent epigenome-wide study [63] has found a strong association between the same CpG site (cg06500161) that we observed and plasma triglycerides. Although the exact mechanism for the implication of this gene in triglyceride metabolism is unclear, it has been posited that the gene modulates bioavailability of plasma lipoprotein lipase and thus induces lipid accumulation in a triglyceride-rich environment [66]. Moreover, another recent epigenome-wide association study identified methylation at *ABCG1* to be significantly associated with waist circumference [67]. It is of interest that we previously observed a strong association of the cg06500161 CpG site with several type 2 diabetes-related traits in this Mexican American cohort [30], and others also found it to be associated with insulin resistance [68]. Thus, these findings raise the possibility that methylation at *ABCG1* may provide critical insights into the reported [69] association of central obesity (HTGW) and type 2 diabetes.

Interestingly, we found a marginally significant association (at the level of the epigenome) between the CpG site cg19693031, in *TXNIP*, and HTGW. The TXNIP protein regulates intra- and extra-cellular reduction-oxidation cycles [70, 71] and genetic variations in this gene have previously been shown to be associated with hypertriglyceridemia in individuals with T2D [72]. Therefore, *TXNIP* also appears to be an attractive candidate involved in the pathogenesis of T2D mediated by HTGW. Future replications and mechanistic studies are required to definitively support the associations observed in this study.

It should be noted that two of the three significantly associated CpG sites contained a SNP within the probe sequence, and it is therefore possible that these SNPs may drive the methylation-HTGW association. However, in a subset of 197 Mexican Americans for whom deep sequencing data was available, we previously found that the SNP was either not present in our cohort subset (for the rs78442314/cg00574958-containing probe), or that it does not significantly influence DNA methylation levels (for the rs9982016/cg06500161-containing probe) [30]. Further, we had observed that association between a SNP and methylation score was much more likely if the SNP was at the CpG site rather than elsewhere within the probe. Together these observations suggest that the CpG sites significantly associated with HTGW in our cohort are unlikely to have been driven by the SNPs contained within the probes and highlights the need for careful consideration of probe-exclusion criteria to avoid the potential loss of important biological associations.

Some limitations of our study need to be considered before generalizing these results. First, our study cannot directly demonstrate the directionality of the methylation ➔ gene expression ➔ phenotype association. However, it has been shown that methylation at cg00574958 can influence expression of *CPT1A* and that methylation within *ABCG1* also influences expression of the gene [22, 63]. Second, DNA methylation is regulated both genetically and environmentally. Since there is a strong environmental correlation between waist circumference and triglycerides, methylation at *CPT1A* and *ABCG1* is associated with HTGW, and HTGW is a significant predictor of type 2 diabetes related traits, our study points towards possible environmental links with known modifiers of methylation such as diet, physical activity, and pharmacological interventions [73, 74]. These possibilities need to be explored further in the future, and may provide stronger justification for putative preventive interventions.

## Conclusions

Prevalence of HTGW and its importance as a marker of T2D has not been clearly demonstrated in Mexican Americans—a high-risk, minority population in the USA. In addition to characterizing HTGW in our study cohort, our results also raise the possibility for an epigenetic basis of HTGW. Our epigenome-wide and genome-wide association studies show that HTGW may be mediated by epigenetic factors, and may also be influenced by the environment. We have identified two CpG sites (cg00574958 and cg17058475) in the 5’ UTR of *CPT1A* and one CpG site (cg06500161) in the body of *ABCG1*, which are associated with HTGW. These genes are involved in *β* oxidation of long-chain fatty acids and triglyceride storage, respectively. Our results highlight the role of epigenetics in HTGW, which is an important marker of T2D and cardiovascular disease.

## Methods

### Study participants

Participants in this study were from the San Antonio Family Heart Study [28, 29], an ongoing prospective evaluation of Mexican American families living in San Antonio. A total of 850 participants from 39 families were included in the analysis. The recruitment and ascertainment of participants and their phenotyping and genotyping have been extensively described elsewhere [29, 75]. This data was collected in the third wave of ascertainment (2002–2006). Peripheral blood samples were collected following an overnight fast and extensive anthropometric phenotyping was conducted.

### Ethics, consent, and permissions

Written consent was obtained for all individuals in this study. This study was approved by the Institutional Review Board at The University of Texas Health Science Center at San Antonio.

### Phenotypes

Our main phenotype of interest was HTGW, which does not presently have a uniform definition across studies [5, 6, 39, 44, 45, 76]. In our study, we defined HTGW as high waist circumference (≥90 cm in males and ≥85 cm in females) combined with high serum triglyceride concentration (≥2.0 mmol/L in males and ≥1.5 mmol/L in females) [5, 33–36]. Other phenotypes included in this study were: age, sex, systolic and diastolic blood pressure, use of anti-lipid, anti-hypertensive and anti-diabetic medications, presence of type 2 diabetes (diagnosed using the ADA criteria [77], fasting glucose ≥7 mmol/L), triglycerides, waist circumference, and presence of obesity (body mass index ≥30 kg/m^2^). Methods used to measure these and other phenotypes have been described previously [29, 75].

### Genotyping

Study participants were previously genotyped for approximately one million single nucleotide polymorphism markers using several Illumina genotyping arrays, including the HumanHap550v3, HumanExon510Sv1, Human1Mv1, and Human1M-Duov3. The Infinium Whole-Genome Genotyping Assay was employed according to manufacturers’ instructions. Details of the data cleaning and imputing steps for this genotypic data have been described previously [20]. Out of a total of 995,320 SNPS genotyped, we incorporated 759,809 SNPs into the association analyses, which had ≥97 % call rate, a minor allele frequency ≥5 % and a Hardy-Weinberg significance value ≥0.001.

### DNA methylation assays and data preprocessing

DNA samples (500 ng) obtained from peripheral blood cells were bisulfite-converted using the EZ-96 DNA Methylation™ Kit (Zymo Research, Irvine, CA) and were subjected to methylation profiling using the Illumina Infinium HumanMethylation450 BeadChip assay (Illumina, San Diego, CA). The array interrogated 485,577 CpG sites across the genome and incorporated both Infinium I and Infinium II bead types. The Illumina iScan was used to scan the BeadChips and raw data was imported into GenomeStudio (V2011.1) to extract image intensities, following background subtraction and normalization to inbuilt controls on the arrays.

Methylation at each CpG site was quantified on a scale from zero (representing fully unmethylated) to one (representing fully methylated) as a methylation score (β). Probes that were located on the sex chromosomes (*n* = 11,648), that were non-CpG loci (*n* = 2,994) or that analyzed SNPs (*n* = 65) were excluded. To correct for differences due to Infinium I and Infinium II probe designs, we used the BMIQ method implemented in the R-based software, BMIQ [78].

We included only those probes for which heritability analyses could be successfully completed without convergence failures. For 12,154 (2.5 %) probes SOLAR was unable to achieve convergence, leaving a total of 458,716 CpG sites available for analysis. Of these, 1385 probes had detection *p* values >0.01 in >5 % of the samples and therefore we excluded these probes leaving a total of 457,331 CpG sites that were finally included in this study. To minimize loss of informative associations, we did not exclude SNP-containing or cross-reactive probes but rather investigated whether the significantly associated CpG sites could have been confounded due to these characteristics.

### Pyrosequencing

For validation of the Illumina microarray data, we performed pyrosequencing on our most significant association (cg00574958 in the *CPT1A*). For each sample, 500 ng of genomic DNA was bisulfite converted, PCR-amplified, and subjected to pyrosequencing with the PyroMark96 MD (Qiagen, Valencia, CA). Percent DNA methylation was determined using PyroMark CpG software 1.0.11.14. The PCR was carried out at 95 °C for 5 min, followed by 40 cycles of 95 °C for 1 min, 56.6 °C for 1 min and 72 °C for 1 min, and a final extension at 72 °C for 7 min. Pyrosequencing was carried out according to the manufacturers’ instructions, and PCR and sequence primers (designed using the Pyromark Assay Design 2.0 software) were designated as

- Forward primer: GTTTTTGGTATTGAGGTAAAATTAA
- Reverse primer (biotinylated): AACCTTTCCAAATTCTTTAAAAC
- Sequence primer: TTTTTGGTATTGAGGTAAAATTAAT

### Statistical analysis

To ensure compatibility with the variance component framework and that the observed associations were unaffected by any undetected skew, we used an inverse normalization preprocessing step for the BMIQ-normalized methylation *β* values to circumvent any distributional aberrations. This step included ranking the raw values, generating cumulative density functions, and determining z-values based on the cumulative densities. All the transformed values were thus distributed as N(0,1) and could be represented on a common, comparable metric of z-values.

Family studies such as the present one have an added advantage that they can shed genetic light on the phenotypes under study. We first estimated the heritability (defined as the proportion of variability explained by genetic similarity among individuals) of HTGW using a polygenic regression model as follows:

$$ l(HTGW)=\mu +\boldsymbol{ba}+{g}_i+{e}_i $$

where *l(HTGW)* is the liability function of HTGW, *μ* is the overall mean liability, ***b*** is the regression coefficient vector corresponding to the covariate matrix ***a***, *g*_*i*_ is the polygenic effect, and *e*_*i*_ is the measurement error. Heritability was then estimated as the ratio of variance due to genetic similarity (modeled as *g*_*i*_ in the equation above) and the total phenotypic variability, *Ω*. The covariates used for the estimation of heritability of HTGW were: age, age^2^, sex, age × sex interaction, age^2^ × sex interaction, use of anti-lipid, anti-hypertensive and anti-diabetic medications, systolic blood pressure, diastolic blood pressure, and presence of type 2 diabetes and obesity (body mass index ≥30 kg/m^2^). Statistical significance of heritability (*H*_o_: heritability = 0) was tested by constraining the heritability to 0 and comparing the likelihood ratio statistics of the constrained and unconstrained models.

For genome-wide association analyses, we used polygenic regression models that accounted for age, age^2^, sex, age × sex interaction, and age^2^ × sex interaction, the top four principal components to quantify ancestry-based population admixture, and use of anti-lipid, anti-hypertensive, and anti-diabetic medications.

Our methylation studies used blood which contains a mixture of cell types. Reinius et al. [79] and Houseman et al. [80] have demonstrated the influence of differential cell proportions on DNA methylation signatures using different array platforms. Jaffe et al. [81] have extended this procedure to the Illumina Infinium HumanMethylation450 array. We estimated the proportion of CD4+ T cells, CD8+ T cells, B cells, natural killer cells, and granulocytes in each sample using the procedure described by Jaffe et al. [81] and adjusted all the polygenic regression models for these estimated cell counts as covariates. To test the association of DNA methylation at each CpG site with the liability function of HTGW, we ran polygenic regression models for each CpG site. In each model, we used age, age^2^, sex, age × sex interaction, and age^2^ × sex interaction, Illumina Sentrix® ID, Sentrix® position, estimated cell counts, and use of anti-lipid, anti-hypertensive, and anti-diabetic medications as covariates. Additional covariates were used for specific analyses and are described in the Results section. In particular, where warranted, we accounted for comorbidities (systolic blood pressure, diastolic blood pressure and presence of type 2 diabetes and obesity) that might influence the HTGW phenotype. Statistical significance for association was tested by constraining the regression coefficient to 0 and comparing the likelihood ratio statistics of the constrained and unconstrained model.

For heritability analyses as well as association analyses, we first estimated the genomic inflation factor (*λ*_median_) which was defined as the median *χ*^2^_LL_/invchi(0.5,1) where invchi(p,d) is the inverse *χ*^2^ function for probability (*p*) and degrees of freedom (*d*), and corrected the nominal significance values for the estimated *λ*_median_. Additionally, we used the Benjamini-Hochberg procedure of false discovery rate (FDR) control for multiple testing correction. Significance was assessed at a global type I error rate of 0.05. The odds ratio (OR) for the association between HTGW and inverse-normalized methylation score was determined as ${e}^{-\sqrt{\pi}\beta }$ where β represents the polygenic regression coefficient. The association of DNA methylation with type 2 diabetes that was mediated through HTGW was estimated using Sobel’s parameter [32]. For this, we ran two regression models for each site—the first model contained HTGW as the outcome and methylation (along with other covariates) as a predictor while the second model used T2D as the outcome and HTGW, methylation and other covariates as predictors. The regression coefficients from these two models were then multiplied to derive a quantified measure of mediation. Standard errors for this parameter were estimated as described by Sobel [32].

To parse out the genetic and environmental covariance, we used the methods entailed under bivariate trait analyses [82–84]. Under this analytical framework, the phenotypic covariance (*ρ*_*P*_^2^) is regarded as a function composed of the additive genetic (*ρ*_*G*_^2^) and environmental (*ρ*_*E*_^2^) covariances between two traits (denoted below as *i* and *j*).

$$ {\rho}_{P^{\left(i,j\right)}}={\rho}_{G^{\left(i,j\right)}}\sqrt{{h_i}^2{h_j}^2}+{\rho}_{E^{\left(i,j\right)}}\sqrt{\left(1-{h_i}^2\right)\left(1-{h_j}^2\right)} $$

These parameters are estimated using an estimation-maximization algorithm by jointly utilizing all available pedigree information with a multivariate normal model for continuous traits and liability threshold model for discrete traits [85–87].

Other statistical methods used were Spearman’s correlation scatter plots and Bland-Altman plots to test for the agreement between different methods of measuring DNA methylation. Heritability, association, and bivariate trait analyses were conducted using the SOLAR software package [88], and all other statistical analyses were conducted using the Stata 12.0 (Stata Corp, College Station, TX) package.

## Additional files

Additional file 1:
**This file contains Supplementary Table 1. ** (XLSX 782 MB) Additional file 2:
**This file contains Supplementary Figs. 1 and 2.** (DOCX 208 KB) Additional file 3:
**This file contains Supplementary Table 2.** (XLSX 314 MB) Additional file 4:
**This file contains Supplementary Table 3.** (XLSX 1982 KB)
