# Supplementary material for: Genome- and epigenome-wide association study of hypertriglyceridemic waist in Mexican American families
Source: Clin Epigenetics. 2016 Jan 20;8:6. doi: 10.1186/s13148-016-0173-x (PMC4721061; doi:10.1186/s13148-016-0173-x)

**Supplementary Figure 1. Q-Q plot for the genome-wide association study of HTGW.**


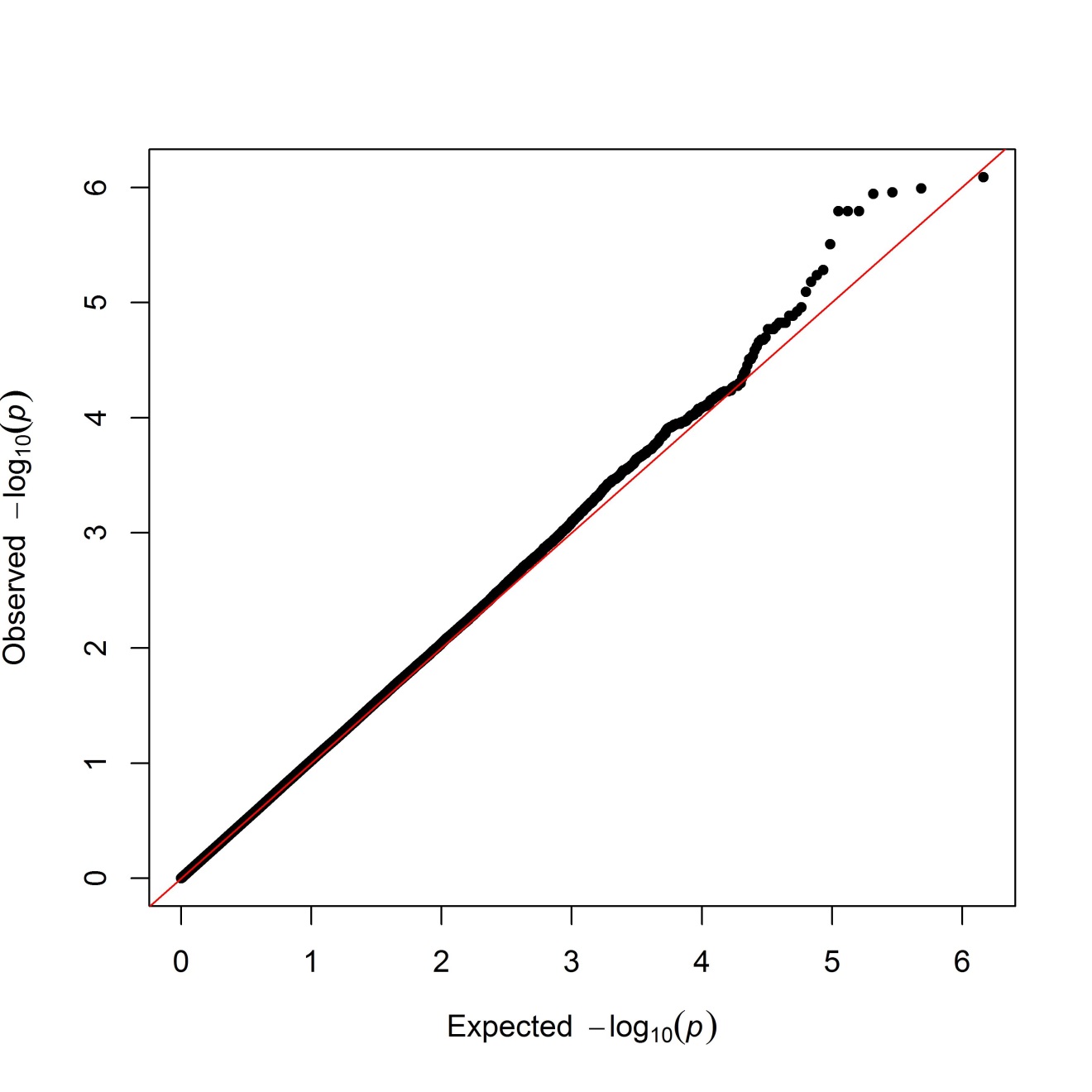


**Supplementary Figure 2. Q-Q plot for the epigenome-wide association study of HTGW.**


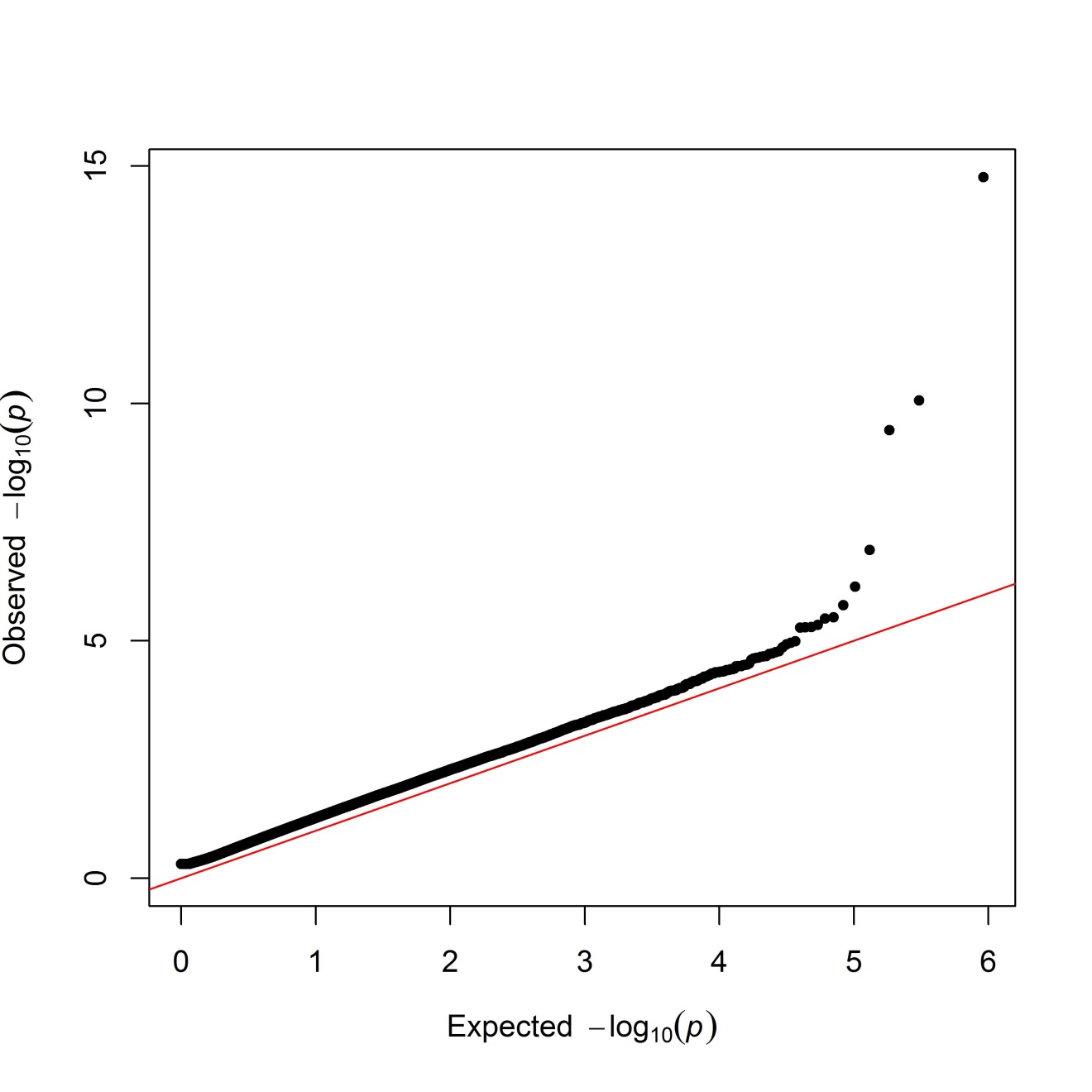

Supplement: Additional file 2: — This file contains Supplementary Figs. 1 and 2. (DOCX 208 KB) [file 13148_2016_173_MOESM2_ESM.docx]
